# Single Nucleotide Polymorphisms of PIN1 Promoter Region and Cancer Risk: Evidence from a Meta-Analysis

**DOI:** 10.1371/journal.pone.0070990

**Published:** 2013-08-16

**Authors:** Jing-Jing Peng, Dong Wei, Dong Li, Zeng-Qiang Fu, Yong Tan, Tao Xu, Jing-Jun Zhou, Tao Zhang

**Affiliations:** Department of Oncology, the General Hospital of Chengdu Military District, Chengdu, Sichuan, P.R. China; Kliniken der Stadt Köln gGmbH, Germany

## Abstract

**Background:**

Peptidylprolyl cis/trans isomerase NIMA-interacting 1 (PIN1) is involved in the process of tumorigenesis. The two single nucleotide polymorphisms (−677T>C, −842G>C) in the PIN1 promoter region have been suspected of being associated with cancer risk for years, but the conclusion is still inconclusive.

**Methods:**

Eligible case-control studies were retrieved by searching databases and references of related reviews and studies. Genotype distribution data, adjusted odds ratios (ORs) and 95% confidence (CIs) intervals were extracted to calculate pooled ORs.

**Results:**

A total of 4619 cancer cases and 4661 controls were included in this meta-analysis. Overall, the PIN1 −667T>C polymorphism was not associated with cancer risk, while the −842C allele was significantly associated with reduced cancer risk (CC+GC vs. GG, OR = 0.725, 95% CI: 0.607–0.865; P_heterogeneity_ = 0.012 and GC vs. GG: OR = 0.721, 95% CI: 0.591–0.880; P_heterogeneity_ = 0.003). Results from genotype distribution data were in agreement with those calculated with adjusted ORs and 95% CIs. No publication bias was detected.

**Conclusions:**

Results of this meta-analysis suggest that the PIN1 −842G>C polymorphism is associated with decreased cancer risk, but that the −667T>C polymorphism is not.

## Introduction

Peptidylprolyl cis/trans isomerase NIMA-interacting 1 (PIN1) belongs to the parvulin peptidyl-prolyl isomerase family. With a conserved WW (Trp-Trp) domain, which is responsible for binding to specific sequences of target proteins and recruiting these proteins into signaling complexes [1], [2]. PIN1 has a high affinity to proteins with Ser/Thr-Pro (Proline) motifs and regulates the conformation of pro-directed phosphorylation sites [2], [3]. Pro-directed phosphorylation is a critical signaling mechanism that regulates various biological processes, such as cell proliferation, differentiation, transcriptional regulation and tumorigenesis [4]. Thus, PIN1 can regulate a lot of signaling pathways including those responsible for tumorigenesis [5] by modulating pro-directed phosphorylation. It has been well demonstrated that numerous oncogenic and tumor suppressor proteins are regulated by PIN1, such as cyclin D1 [6], c-Jun [7], Bcl-2 [8], β-catenin [9] and p53 [10]. Therefore, PIN1 functions as a critical regulator during the process of tumorigenesis.

Consistent with the critical regulatory role of PIN1 in cancer development, it has been reported that aberrant expression of PIN1 is associated with cancers, such as breast cancer [7], [11] and prostate cancer [12]. Additionally, studies suggest that single nucleotide polymorphisms (SNPs) of PIN1 are associated with Alzheimer's disease [13], [14] and cancer [15]–[22]. The two SNPs (−667T>C rs2233679, −842G>C rs2233678) in the promoter region of PIN1 have been mostly investigated. In 2007, Segat and colleagues [21] found the −667T>C polymorphism was associated with hepatocelluar carcinoma (HCC) that was co-infected with hepatitis B virus and hepatitis B virus; however, other researchers did not found any significant association of THE −667T>C polymorphism with other cancers [16], [17]. On the other hand, Han et al [16] showed that the C allele of the −842G>C polymorphism could reduced the risk of breast cancer, while Naidu suggested that the −842G>C polymorphism did not affect susceptibility to breast cancer [20]. Briefly, the association of the two SNPs of PIN1 (−667T>C, −842G>C) with cancer risks is elusive according to current literatures.

The present meta-analysis was designed to ascertain whether the two common SNPs (−667T>C, −842G>C) of PIN1 are associated with cancer risk and evaluate the impact of ethnicities.

## Methods

### Searching strategy

Databases of PubMed, EMBASE and China National Knowledge Infrastructure (CNKI) were searched to retrieve eligible case-control studies. Key words of “peptidylprolyl cis/trans isomerase, NIMA-interacting 1”, “single nucleotide polymorphism”, and “cancer” were used and the alternative spellings of these key words were also considered. There was no limitation of search and the last search was performed on March, 2013. Reference lists of related studies and reviews were manually searched for additional studies.

### Inclusion and exclusion criteria

Eligible studies were identified according to the following inclusion criteria: (1) case-control studies; (2) investigating the correlation between the −842G>C or −667T>C polymorphism and cancer risk; (3) providing detail genotype frequencies. Studies without detail genotype frequencies were excluded. Searching records were primarily searched by titles and abstracts and then full text articles were retrieved for further evaluation of eligibility. Two reviewers (PJJ and WD) extracted eligible studies independently according to the inclusion criteria. Disagreement between two authors was discussed with another author (ZT) till consensus was achieved.

### Data Collection

Data of eligible studies was extracted by two authors (PJJ and WD) independently in duplicate with a predesigned data-collection form. The following data was collected: name of first author, year of publication, country where the study was conducted, genotyping methods, ethnicity, cancer type, source of control, number of cases and controls, genotype frequency in cases and controls, adjusted odds ratios (ORs) and 95% confidence intervals (CIs). In this meta-analysis, ethnicity was simply categorized as Asian and Caucasian. Source of control was defined as hospital-based (HB) and population-based (PB) according to the control source. Sample size of studies were defined as large (>500 participants) or small (≤500 participants). In the study by Segat et al [21], no information about control source was available, thus we classified this study as HB. Two reviewers reached consensus on each item.

### Statistical analysis

To test the distribution of Hardy-Winberg equilibrium (HWE) in controls, chi-square test for goodness of fit was conducted and a p<0.05 indicated disequilibrium of HWE. We assessed the association strength of the PIN1 −667T>C and −842G>C polymorphisms with cancer risk by OR and 95% CIs. The 95% CIs was used for statistical significance test and a 95% CI without 1 indicating a significantly increased or decreased cancer risk. We calculated pooled ORs for homozygote comparison (CC vs. TT or GG), heterozygote comparison (TC vs. TT and GC vs. GG), dominant model (TC+CC vs. TT or GC+CC vs. GG) and recessive (CC vs. GC+GG or TC+TT) model, assuming the dominant and recessive effect of the variant C allele, respectively. We performed subgroup analyses to explore the effects of confounding factors, such as ethnicities, source of control and sample size. By omitting one study each time, sensitivity analyses were performed to investigate individual study' effect on pooled results and test the reliability of meta-analysis results.

Chi-square based Q test was utilized to assess the statistical heterogeneity between studies, and heterogeneity was significant when p<0.10. The fixed-effects model (based on Mantel-Haenszel method) and random-effects model (based on DerSimonian-Laird method) were used to calculated the pooled ORs. The fixed-effects model was used when there was no significant heterogeneity; otherwise, the random-effects model was applied [23]. Meta-regression was performed to detect the source of heterogeneity, and a p<0.05 was considered significant.

Begg's funnel plot and the Egger' linear regression test were conducted to detect publication bias, and a p<0.05 was considered significant [24]. All statistical analyses were conducted with STATA software (version 10.0; StataCorp, College Station, Texas USA). And all p values are two-side.

## Results

### Identification of eligible studies

**Figure 1:**
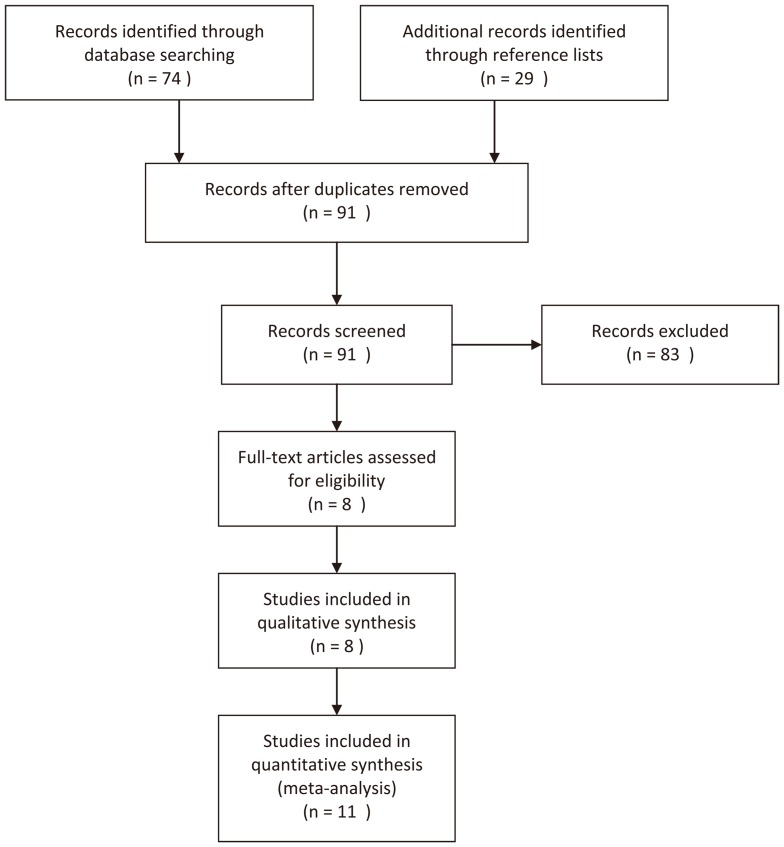
Flow Diagram. *Data from Lu [18] were treated as 2 studies, and data from Naidu [20] were treated as 3 studies.

After removal of duplicated records, a number of 91 searching records were screened and 8 full-text articles [15]–[22] were retrieved after primary screening. In the study by Naidu et al [20], they investigated three populations and the genotype distribution were reported separately, therefore, the three populations were regarded as 3 studies; Lu also reported data of test set and validation set independently [18], and the two sets were treated as 2 studies. Thus, 11 studies were included in the quantitative synthesis. The detailed screening process was shown in Figure 1.

### Characteristics of included studies

A total of 11 studies were included in this meta-analysis, including 4619 cancer patients and 4661 controls. PIN1 polymorphisms and cancer risk was investigated in 7 kinds of cancer (hepatocellular carcinoma, laryngeal squamous cell carcinoma, squamous cell carcinoma of the head and neck, breast cancer, lung cancer, esophageal carcinoma and nasopharyngeal carcinoma). Polymerase chain reaction–restriction fragment length polymorphism (PCR-RFLP) was used for genotyping in most studies. The detailed characteristics of eligible studies are shown in Table 1. Genotype distributions of the −667T>C and −842G>C polymorphism in controls were in agreement with HWE, except for the −842G>C polymorphism in the study reported by Segat and colleagues [21].

**Table 1:** Characteristics of 11 eligible studies.

Author	Country	Ethnicity	Cancer	Control	Cancer Cases			Controls			HWE	
					No.	Age(year)	Male	No.	Age	Male	−667T>C	−842G>C
Lu Y	China	Asian	NC	HB	178	46.1+11.1^a^	69.1%	156	44.5+9.9	67.9%	0.06	0.06
You Y	China	Asian	EC	PB	699	>58,50.1%	83.4%	729	>58,48.4%	81.2%	0.58	0.31
Lu Ja†	China	Asian	LC	HB	1056	>60,49.2%	70.6%	1056	>60,49.4%	70.6%	0.63	0.89
Lu Jb†	China	Asian	LC	HB	503	>60,45.7%	66.6%	623	>60,44.9%	70.4%	0.92	0.52
Naidu R(Malay)‡	Malaysia	Asian	BC	PB	387	53.1+11.7^ a^	0	252	52.3+11.3	0	0.92	0.89
Naidu R(Chinese)‡	Malaysia	Asian	BC	PB							0.08	0.92
Naidu R(Indian)‡	Malaysia	Asian	BC	PB							0.75	0.2
Han CH	USA	Caucasian	BC	HB	467	>45,57.4%	0	488	>45,56.6%	0	0.23	0.16
Lu J	USA	Caucasian	SCCHN	HB	1006	56.9+11.3^ a^	77.1%	1007	57.4+11.4	77.2%	0.08	0.64
Cao WP	China	Asian	LSCC	HB	95	63.25+11.33^ a^	77.9%	100	60.23+11.12	71%	0.35	0.47
Segat L	Italy	Caucasian	HCC	HB	228	NA	NA	250	NA	NA	0.85	0.01*

HB: hospital-based; PB: population-based; PCR-RFLP: polymerase chain reaction-restricted fligment length polymorphism; HWE: Hardy-Winberg Equibrilium; NA: not available; NC: nasopharyngeal carcinoma; EC: esophageal carcinoma; LC: lung cancer; BC: breast cancer; SCCHN: squamous cell carcinoma of the head and neck; LSCC: laryngeal squamous cell carcinoma; HCC: hepatocellular carcinoma; a: mean + standard devation; † the study by Lu J [18] was treated as two studies, Lu Ja: test set, Lu Jb: validation set;‡ in the study by Naidu R [20], each population was considered as a study.

### −677T>C polymorphism and cancer risk

**Figure 2:**
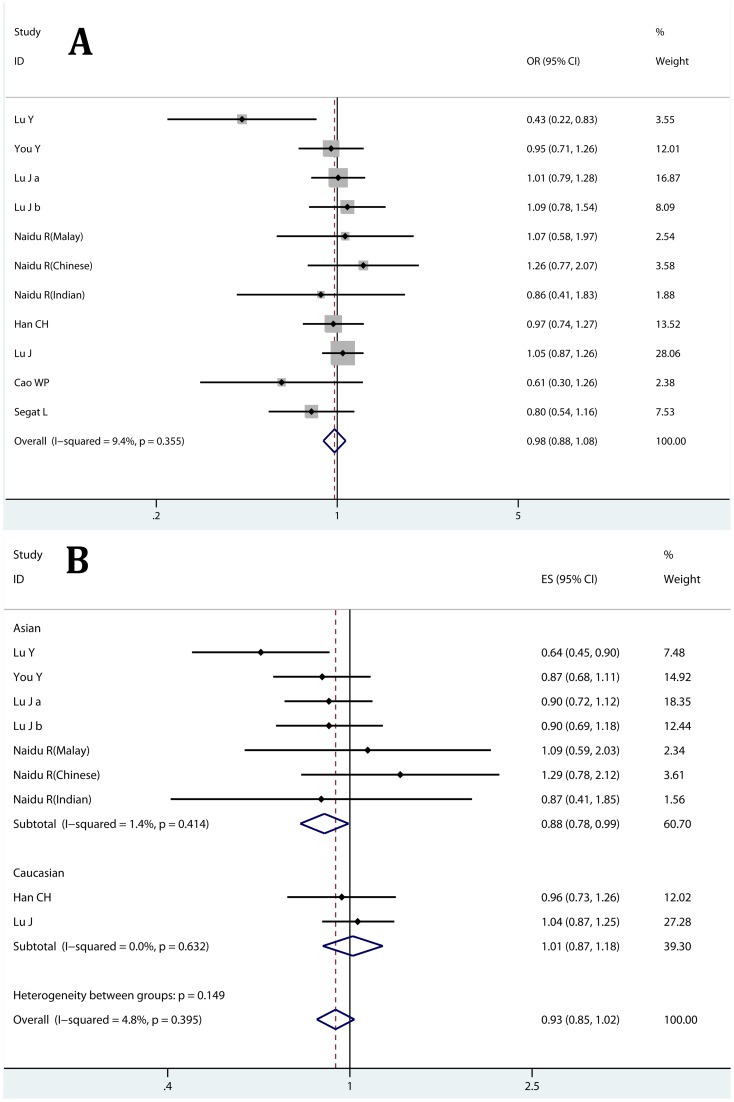
Correlation between the PIN1 −667T>C polymorphism and cancer risk. A: heterozygote comparison (TC vs. TT) estimated with genotype distribution data; B heterozygote comparison (TC vs. TT) calculated with adjusted ORs and 95% CIs; Lu J a: test set [18]; Lu J b: validation set [18].

By pooling data of genotype distribution, in overall comparison, no significant association of the −667T>C polymorphism with cancer risk was found in any comparison model (Figure 2A; Table 2). Subgroup analyses were conducted to further evaluate the impact of ethnicities, sources of control and sample size. Results suggested that there was no significant association of the −667T>C polymorphism with cancer risk in any of the subgroups (Table 2). Notably, no significant heterogeneity among studies was detected (Table 2). Sensitivity analysis revealed that no individual study affected the pooled results significantly (data not shown).

**Table 2:** Association of PIN1 polymorphisms with cancer risk estimated with raw data.

	Homozygote comparison		Heterozygote comparison		Recessive Model		Dominant Model	
	OR(95% CI)	P	OR(95% CI)	P	OR(95% CI)	P	OR(95% CI)	P
−667T>C, rs2233679								
Overall	1.036(0.912–1.178)	0.58	0.976(0.884–1.078)	0.355	1.079(0.977–1.191)	0.927	0.996(0.907–1.095)	0.384
HB	1.025(0.885–1.186)	0.235	0.967(0.865–1.081)	0.135	1.065(0.950–1.194)	0.777	0.986(0.887–1.096)	0.128
PB	1.077(0.826–1.404)	0.946	1.011(0.811–1.261)	0.761	1.123(0.918–1.375)	0.814	1.039(0.844–1.278)	0.902
Asian	1.051(0.901–1.227)	0.563	0.964(0.838–1.109)	0.232	1.097(0.981–1.227)	0.936	1.001(0.878–1.143)	0.334
Caucasian	1.005(0.799–1.263)	0.273	0.988(0.858–1.138)	0.428	1.013(0.815–1.258)	0.445	0.991(0.866–1.134)	0.263
Large	1.106(0.961–1.273)	0.966	1.016(0.909–1.135)	0.954	1.102(0.990–1.227)	0.959	1.042(0.938–1.159)	0.954
Small	0.754(0.553–1.030)	0.657	0.831(0.664–1.039)	0.156	0.951(0.733–1.235)	0.743	0.833(0.675–1.029)	0.243

−842G>C, rs2233678								
Overall	0.740(0.515–1.063)	0.252	0.721(0.591–0.880)*	0.003	0.800(0.559–1.146)	0.177	0.725(0.607–0.865)*	0.012
HB	0.900(0.601–1.348)	0.28	0.701(0.535–0.919)*	0.001	0.978(0.656–1.460)	0.19	0.728(0.574–0.924)*	0.003
PB	0.315(0.129–0.769)*	0.925	0.714(0.559–0.910)*	0.378	0.332(0.136–0.808)*	0.952	0.677(0.538–0.853)*	0.425
Asian	0.768(0.486–1.212)	0.117	0.641(0.543–0.757)*	0.33	0.849(0.540–1.335)	0.09	0.654(0.559–0.764)*	0.35
Caucasian	0.695(0.384–1.261)	0.627	0.926(0.572–1.499)*	0.001	0.725(0.401–1.310)	0.483	0.892(0.589–1.353)*	0.004
Large	0.706(0.436–1.142)	0.868	0.676(0.597–0.765)*	0.897	0.757(0.468–1.223)	0.855	0.677(0.599–0.765)*	0.868
Small	0.787(0.454–1.365)	0.048	0.779(0.455–1.333)*	<0.001	0.860(0.500–1.479)	0.029	0.789(0.501–1.243)*	0.003

OR: odds ratio; P: p value of heterogeneity; HB: hospital-based; PB: population-based; *significant association.

To better assess the effect of PIN1 polymorphisms, we calculate pooled OR with adjusted ORs and 95% CIs. Similarly, we found the PIN1 −667T>C polymorphism did not contribute to cancer risk in overall comparison (Figure 2B; Table 3). However, Asian carriers of the −667TC genotype may have a reduced risk of cancer (Heterozygote comparison TC vs. CC: OR = 0.880, 95% CI: 0.779–0.993; P_heterogeneity_,0.414; Table 3), while this was not observed in Caucasian population. Heterogeneity was not significant among studies and no individual study significantly affected the pooled results.

**Table 3:** Association of PIN1 polymorphisms with cancer risk estimated with adjusted ORs and 95% CIs.

	Homozygote comparison		Heterozygote comparison		Dominant Model	
	OR(95% CI)	P	OR(95% CI)	P	OR(95% CI)	P
−667T>C, rs2233679						
Overall	1.100(0.957–1.265)	0.491	0.931(0.847–1.023)	0.395		
HB	1.093(0.929–1.285)	0.133	0.925(0.831–1.030)	0.187		
PB	1.122(0.853–1.477)	0.95	0.949(0.777–1.159)	0.535		
Asian	1.105(0.933–1.308)	0.323	0.880(0.779–0.993)*	0.414		
Caucasian	1.091(0.851–1.398)	0.503	1.015(0.873–1.180)	0.632		
Large	1.152(0.994–1.335)	0.938	0.946(0.853–1.048)	0.766		
Small	0.735(0.475–1.138)	0.396	0.849(0.665–1.084)	0.115		

−842G>C, rs2233678						
Overall	0.589(0.394–0.880)*	0.885	0.664(0.590–0.747)*	0.527	0.668(0.594–0.751)*	0.638
HB	0.682(0.435–1.069)	0.901	0.665(0.582–0.761)*	0.681	0.678(0.593–0.776)*	0.812
PB	0.327(0.133–0.804)*	0.905	0.660(0.516–0.842)*	0.188	0.636(0.502–0.806)*	0.26
Asian	0.486(0.292–0.810)*	0.896	0.632(0.542–0.738)*	0.442	0.636(0.545–0.742)*	0.547
Caucasian	0.806(0.419–1.550)	0.956	0.710(0.592–0.850)*	0.55	0.713(0.596–0.853)*	0.6
Large	0.720(0.442–1.173)	0.938	0.656(0.578–0.745)*	0.682	0.656(0.580–0.743)*	0.663
Small	0.386(0.190–0.784)*	0.837	0.715(0.521–0.982)*	0.208	0.768(0.537–1.098)	0.346

OR: odds ratio; P: p value of heterogeneity; HB: hospital-based; PB: population-based; *significant association.

### PIN1 −842G>C polymorphism and cancer risk

**Figure 3:**
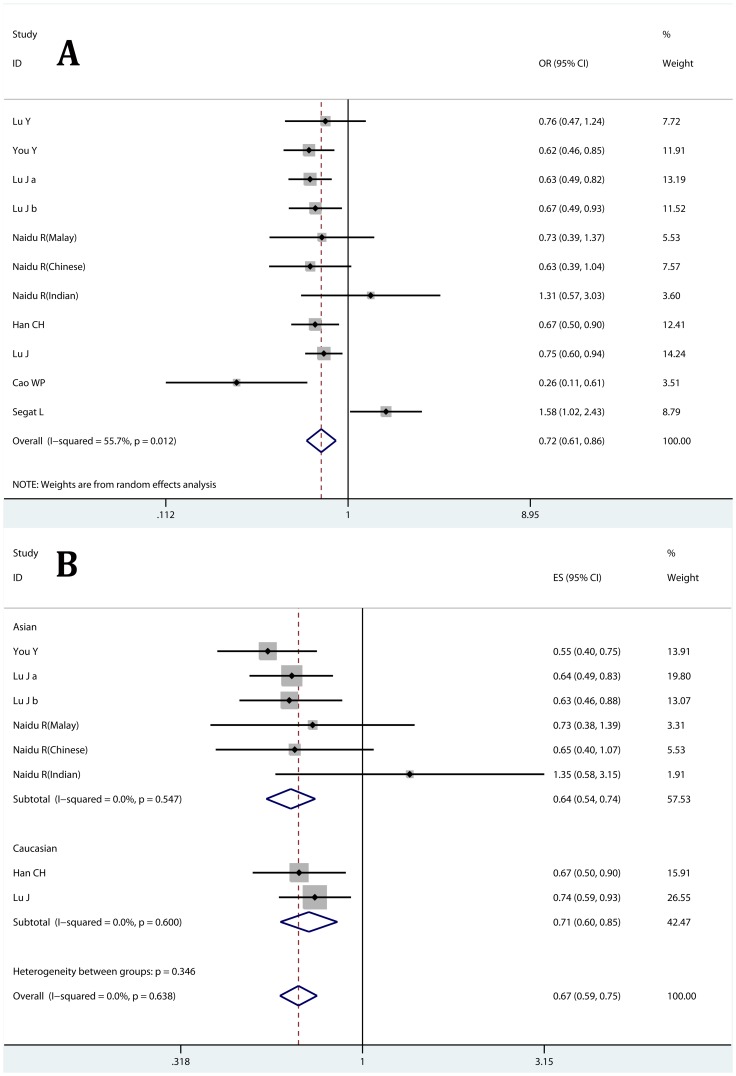
Association of PIN1 −843G>C polymorphism with cancer risk. A: dominant model (GC+CC vs. GG) calculated with genotype distribution data; B dominant model (GC+CC vs. GG) calculated with adjusted ORs and 95% CIs; Lu J a: test set [18]; Lu J b: validation set [18].

Meta-analysis results from genotype distribution data showed that the −842C allele could significantly reduced cancer risk (Dominant model CC+GC vs. GG: OR = 0.725, 95% CI: 0.607–0.865; P_heterogeneity_ = 0.012 Figure 3A; and Heterozygote comparison: GC vs. GG: OR = 0.721, 95% CI: 0.591–0.880, P_heterogeneity_ = 0.003; Table 2). The decreased cancer risk was also confirmed subgroup analyses (Table 2). Sensitivity analysis suggested no individual study affected pooled result significantly, while heterogeneity among studies was observed.

When assessing the −842G>C polymorphism and cancer risk using adjusted ORs and 95% CIs, we found the −842C allele was associated with a decreased risk in all 3 comparison models and most subgroups (Figure 3B; Table 3). Additionally, no significant heterogeneity was observed and no individual study affected the pooled results.

### Publication bias and meta-regression

**Figure 4:**
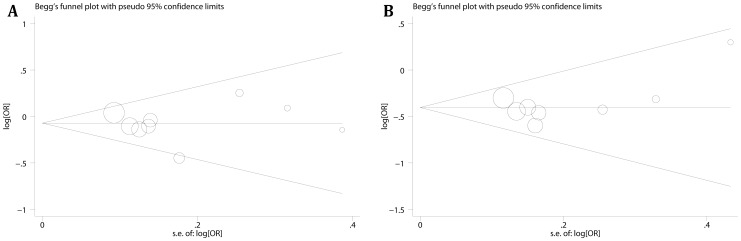
Funnel plots for −667T>C and −842G>C polymorphism. A: heterozygote comparison (TC vs. TT) of −667T>C polymorphism estimated with adjusted ORs and 95% CIs, P_Begg_ = 0.917, P_Egger_ = 0.89; B: dominant model (GC+CC vs. GG) of −842G>C polymorphism calculated with adjusted ORs, P_Begg_ = 0.536, P_Egger_ = 0.366.

Publication bias were evaluated by Egger's test and Begg's test, and we did not found any evidence of publication bias (Figure 4, heterozygote comparison of −667T>C and dominant model of −842G>C were given for example). To search the source of heterogeneity, meta-regression was performed. Cancer types (p = 0.003), ethnicities (p = 0.001), sources of control (p = 0.006) and sample size (p = 0.001) were the sources of heterogeneity. Additionally, study by Segat [21] also contributed to heterogeneity (p = 0.015), and no significant heterogeneity (Heterozygote comparison, P_heterogeneity_ = 0.418; Dominant model, P_heterogeneity_ = 0.455) was observed after removal of this study.

## Discussions

Human PIN1 gene, located on chromosome 19p13, contains 4 exons and has a promoter region of about 1.5 kb. The −667T>C and −842G>C polymorphisms occur in PIN1 promoter region, and they have been suspected as risk factors of cancer [16], [17], [21] and Alzihamer's disease [13], [14]. Recently, data from a meta-analysis showed that neither the −667T>C nor −842G>C polymorphism was associated with susceptibility to Alzihamer's disease [25]; however, the correlation between PIN1 polymorphisms and cancer risk is still inconclusive.

In this meta-analysis, we found the −667T>C polymorphism did not contribute to susceptibility to cancer, while the −842C allele was significantly associated with reduced risk of cancer. To better evaluate the effect of PIN1 polymorphisms, adjusted ORs and CIs from eligible studies were collected and analyzed. These findings were also supported by results from adjusted ORs. Additionally, results from adjusted ORs also suggested that carriers of the −667TC genotype may have a decreased cancer risk in Asian.

Lu and colleagues [17] have demonstrated that the −842C allele of PIN1 is associated with reduced transcriptional activity. Combined with the oncogenetic role of PIN1 [18], this may explain why the −842C allele reduced cancer risk. Segat first reported that −667T allele was associated with increased risk of HCC co-infected with HBV and HCV [21], while subsequent studies did not found any differences between the −667T and −667C allele [3], [13], [24]. In the study by Segat [21], a set of highly selected patients were included, and they could not represent all HCC. In addition, the genotype distribution of −842G>C was in disagreement with HWE, which may be the reason why they found an increased risk.

Heterogeneity was observed in the heterozygote comparison and dominant model of −842G>C polymorphism. The heterogeneity among studies was caused by cancer types, sources of control, ethnicities and sample size. Additionally, in terms of individual study, Segat's study [21] contributed to heterogeneity, but sensitivity analysis revealed that this study did not affect the pooled results significantly. Thus, results from our meta-analyses were robust and reliable.

It has been well documented that meta-analysis increases in statistical power [26] and various studies have validated the efficacy of meta-analysis [27], [28]. In this meta-analysis, we included 4619 cancer cases and 4661 controls, which can provide enough statistical power. We also calculated pooled ORs with adjusted ORs and CIs, which could more precisely reflect gene effect. Also, limitations of this study should be noted. This meta-analysis was based on studies of different kinds of cancer, thus it should be caution to interpret our results. Because of the varied etiology of cancer, these 2 SNPs of PIN1 may have different functions according to cancer types. Additionally, due to limited number of studies, we did not performed subgroup analyses according to cancer types.

To summary, we demonstrate that the −667T>C polymorphism of the PIN1 do not contribute to cancer risk, while the −842C allele is associated with reduced cancer risk.

## Supporting Information

Checklist S1 **PRISMA checklist.**
(DOC)
